# Health Tract, No. 272

**Published:** 1868-03

**Authors:** 


### HEALTH TRACT, No. 272.

### TYPOHID.

If you knock a man down, he may rise up again, but after two or three such knockings he loses the power of rising. In ordinary fevers the system has a recupurative power, especially when the weight of the malady has been removed by suitable medicine ; but when that recupurative power is lost, the system will not rise to health, although medicine has done all that was expected from it, and the patient dies. This inability may exist in all forms of disease. "Typhoid" means "like typhus," and typhus itself means "stupor" — a kind of sleep or death. There is a growing tendency in all diseases "to take on the typhoid type," which simply means that the constitutions of the people are growing weaker and weaker, less and less capable of resisting the onsets of disease; hence a less amount of sickness kills now, than formerly; and added to this, physicians of every grade have observed that their patients 'can't bear' as large doses of medicine as heretofore, and the tendency is to give less, and at longer intervals and wait and see "what nature will do." The practical use to be made by the reader of these facts is to habituate himself to a greater watchfulness against the causes of all disease, and to a greater care of himself when he is sick ; and this care should be observed in three main directions—

1. In recovering from any form of disease, keep abundantly and comfortably warm.
2. Studiously avoid taking cold.
3. Watch against over-exercise for several days or weeks.
4. Eat very moderately and at regular intervals, of plain, nourishing food.

If these four things are observed, relapses wouldbe rare, and the patient would be saved ; the most difficult of the four, is to avoid eating too much ; there is special danger of yielding to a craving for some particular kind of food. We knew an estimable lady who was happily recovering from an attack of typhoid fever, but she had such a strong desire for a sweet potatoe that it was allowed her; in less than an hour the symptoms became unfavorable and she died the next day.

The sleepiness or stupor of typhoid arises from the fact that the brain, and thence the whole nervous system, is oppressed by the disease ; is weighed down; can't act; — goes to sleep and dies!

